# Anomaly Detection in Nanofibrous Materials by CNN-Based Self-Similarity

**DOI:** 10.3390/s18010209

**Published:** 2018-01-12

**Authors:** Paolo Napoletano, Flavio Piccoli, Raimondo Schettini

**Affiliations:** Department of Computer Science, Systems and Communications, University of Milano-Bicocca, Milan 20126, Italy; flavio.piccoli@disco.unimib.it (F.P.); schettini@disco.unimib.it (R.S.)

**Keywords:** anomaly detection, defect detection, industrial quality inspection, quality control, convolutional neural networks, nanofibrous materials

## Abstract

Automatic detection and localization of anomalies in nanofibrous materials help to reduce the cost of the production process and the time of the post-production visual inspection process. Amongst all the monitoring methods, those exploiting Scanning Electron Microscope (SEM) imaging are the most effective. In this paper, we propose a region-based method for the detection and localization of anomalies in SEM images, based on Convolutional Neural Networks (CNNs) and self-similarity. The method evaluates the degree of abnormality of each subregion of an image under consideration by computing a CNN-based visual similarity with respect to a dictionary of anomaly-free subregions belonging to a training set. The proposed method outperforms the state of the art.

## 1. Introduction

Cyber-Physical systems [1], Internet of Things [2], Cloud Computing [3] and Cognitive Computing [4] are the key ingredients of the fourth industrial revolution that will lead to development of the so-called Industry 4.0 [5,6]. The industry of the future is a “smart factory” that integrates new hardware, software and communication technologies to obtain smart production processes that increase productivity and reduce costs in manufacturing environments. One of the challenges of the Industry 4.0 designers is the optimization of the manufacturing processes. A key element to this aim is the early, or production-compatible-time, detection of defects and production failures. This enables the prevention of production errors, thus increasing productivity, quality, and consequently leading to a economic benefit for the factory [5].

Defect detection in industry is performed in several ways: (1) manually by humans that monitor the entire production process; (2) automatically by a computer-based system that monitors, mostly with the help of digital cameras or sensors, the production process; (3) semi-automatically by humans that interact with a computer-based defect detection system [7,8,9,10]. In all ways, defect detection is performed in two different moments: during or at the end of the production process. To take the chance to correct the production process, defect detection should be performed during the production process in a time that should be less, or at most comparable with, the production time itself. This time constraint permits providing feedbacks or alarms that may be used to correct the production process [7].

In this paper, we focus on the automatic detection of defects from images. In particular, we focus on the detection of defects in nanofibrous materials through the analysis of Scanning Electron Microscope (SEM) images. Nanofibrous materials are fibers with diameters smaller than 100 nanometers that are largely employed in several application domains [11,12], such as sensors [13,14], life science and medicine [15,16], filtration and water treatment [17,18], etc. Nanofibers are usually composed of biopolymers (such as gelatine, chitosan, collagen, etc. [19,20]), synthetic polymers, both organic and inorganic (such as polystyrene, polyamidimide, polyacrylonitrile, polysiloxanes, etc.) [21,22], and metallic polymers such as silver, gold, manganese, etc. [23].

**Figure 1:**
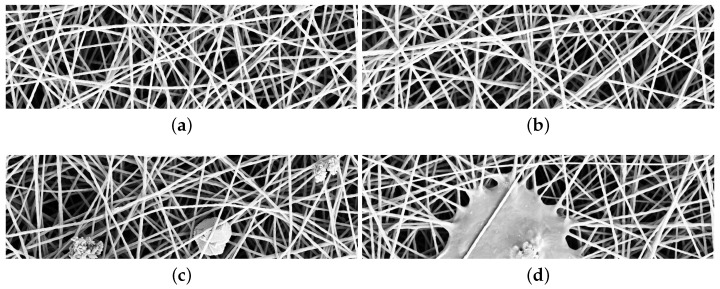
SEM images of nanofibrous materials. (**a**,**b**) samples without anomalies; (**c**,**d**) samples containing fine- and coarse-grained anomalies.

The most common method used to produce nanofibrous materials is the electrospinning [24]. The production process of nanofibers, due to several variables, such as applied voltage and polymer concentration, temperature and relative humidity, etc., may generate structural defects. Defects are usually beads or flattened areas, and have non-uniform distributions, such as alternation of big pores and dense material, and sparse fiber diameter distribution. Automatic detection of defects in nanofibrous materials would reduce both the cost of production process and the time spent in quality inspection. Scanning Electron Microscope images have a resolution up to one nanometer and thus they may be used to detect and localize both fine- and coarse-grained defects [25,26,27]. Figure 1a,b contains examples of SEM images without defects. Figure 1c contains examples of fine-grained defects: a small speck of dust at the center and two beads (fiber clots) on the right and left side. Figure 1d contains an example of coarse grained defect: a thin layer of material among the fibers, that is a film.

The methods for monitoring the nanofiber production process are divided into two categories. The first class includes methods that check for the functional properties of the nanofibers, for example as filtering behavior in the case of filters [17,28]. The methods usually require a high executing time and thus are inadequate for generating alarms in a short time. The second class includes methods that automatically check the structure of the nanofibrous material through computer-vision-based inspection of SEM images [25]. These methods do not require high computational time, thus allowing for generating alarms in a short time during the production process [25].

An automatic computer-vision-based defect detection method can be reference or no-reference-based [7]. In the first case, each item with a given type of defect as well as items without defects are modeled. In the case of the no-reference-based one, there is no a priori knowledge about items with defects but only knowledge about items without defects. For no-reference based methods, defects are detected as anomalies with respect to normal items; in other words, anomalies are items that do not conform to an expected pattern. From the machine-learning point of view, reference-based methods are implemented through a multi-class classifier while no-reference-based methods are implemented through a one-class classifier that takes “normal” (anomalies-free) images for training, and “normal” and “anomalous” images for testing.

In this paper, we propose a no-reference method for automatic detection and localization of anomalies within SEM images of nanofibrous materials. The proposed method is region-based; it measures how much a subregion of the test image is anomalous with respect to a dictionary composed of “normal” subregions taken from the training images. The degree of abnormality of a subregion is measured as the visual similarity between a testing subregion and a “normal” subregion of the dictionary. The lower is the similarity between two subregions and the higher is the degree of abnormality, that is, how much a subregion is anomalous. The visual similarity between two subregions is the Euclidean distance between the feature vectors extracted from the two subregions. The feature vectors are extracted by exploiting a Convolutional Neural Network. A subregion is classified as anomalous if its degree of abnormality is higher than a given threshold found by estimating the distribution of the visual self similarity among subregions taken from “normal” images. Since the proposed method is region based, it permits detecting and localizing the defects at the same time. The precision in defect localization is a trade-off between the size of the subregion and spatial overlap between subsequent subregions. The novel contributions of this paper are mainly two: the proposed method, differently from the state of the art [25], uses for training and tuning the parameters only “normal” images, and it exploits a general purpose (and not an ad hoc one) Convolutional Neural Network for describing the visual content of each subregion of SEM images. From the experimental point of view, we also investigate the trade-off between computational time and performance.

Performance, measured with the area under the curve and percentage of defect area coverage, demonstrates that the proposed method outperforms the state of the art. The computational time required to process a test image is comparable with the production time of nanofibrous materials, thus demonstrating that this method can be employed for monitoring the production process as well as for the visual inspection of nanofibers.

## 2. Related Works

Anomaly detection is a problem largely investigated in literature within several research areas such as computer vision, signal processing, communications, etc. [29], and within several application domains such as industrial damage detection [7], texture defect detection [30], medical anomaly detection [31], textual anomaly detection [32], sensor networks [33], etc.

We can group anomaly detection techniques in five categories: probabilistic, distance-based, reconstruction-based, domain-based, and information-theoretic techniques [34]. Probabilistic methods assume that low density areas in the training set have a low probability of containing “normal” objects. These methods try to estimate the density of the “normal” class and then use this density to estimate the probability of a test pattern of being “anomalous”. Distance-based methods are based on the assumption that “normal” data are tightly clustered while anomalous data are from the “normal” ones. These methods consider the use of nearest-neighbours and data clustering techniques. Reconstruction-based methods consider a regression model trained on “normal” data. Anomalous patterns are discovered, estimating the error between the actual regression value and the regression target obtained with “normal” data during the training phase. Domain-based methods, in a way similar to distance-based methods, try to describe a domain containing “normal” data by defining a boundary around the “normal” class. The detection of anomalies is then obtained by checking whatever or not a test pattern falls within the “normal” class. The last category of methods is the information-theoretic one. These methods compute the information-theoretic measure, such as entropy or Kolmogorov complexity, for “normal” images and measure how much a test pattern alters the information content of “normal” images. Readers who would wish to deepen the subject can refer to [29,34].

Concerning detection of anomalies within SEM images of nanofibrous materials, a recent method has been proposed by Carrera et al. [25]. This method is based on a dictionary yielding sparse representation built by following a previous work proposed by Boracchi et al. [35]. The dictionary represents normal data and it is used in a test phase to detect anomalies in a patch-wise manner. Anomalies are then identified by quantitatively assessing whether the patch conforms or not to the learned model. The Carrera et al. method outperformed the other methods in the state of the art, such as a distance-based method that uses structural texture similarity metrics (STSIM) to detect anomalies [36] and a method that learns a model of normal data based on a sparse representation [37]. For these reasons, we will compare our proposed method with their one.

## 3. Problem Formulation

Let us define an image I as a matrix of size w×h×c of values ∈N. The variable *h* is the height, *w* is the width and *c* is the number of color channels, which is equal to 3 in the case of Red, Green and Blue (RGB) images. Each element or pixel of the image I(p) at position *p* within the matrix of size w×h, is a triplet of RGB values ∈N. Let us define $\Omega_{I}$ as the binary mask of anomalies for the image I. $\Omega_{I}$ is a matrix of size w×h and each element at position *p* is such that:

(1) $$\Omega_{I}\left(p\right)=\left\{\begin{array}{cc} 0, & \mathrm{if}\;I(p)\;\mathrm{is}\;\mathrm{not}\;\mathrm{an}\;\mathrm{anomalous}\;\mathrm{element}\;\mathrm{of}\;\mathrm{the}\;\mathrm{image}\;I,\\ 1, & \mathrm{if}\;I(p)\;\mathrm{is}\;\mathrm{an}\;\mathrm{anomalous}\;\mathrm{element}\;\mathrm{of}\;\mathrm{the}\;\mathrm{image}\;I. \end{array}\right.$$

**Figure 2:**
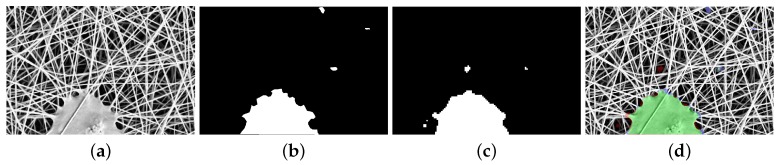
(**a**) the input image I; (**b**) the binary mask of anomalies $\Omega_{I}$. White pixels represent anomalies; (**c**) estimated mask of anomalies $\tilde{\Omega_{I}}$. White pixels represent anomalies; (**d**) difference between $\Omega_{I}$ and $\tilde{\Omega_{I}}$ overlaid on the test image. Green pixels represent *true positives*, red pixels represent *false positives*, blue pixels represent *false negatives*, and no color pixels represent *true negatives*.

Given an image I and its mask of anomalies $\Omega_{I}$, the anomaly detection problem is defined as the problem of automatically finding the binary mask $\tilde{\Omega_{I}}$ that best approximates the reference mask of anomalies $\Omega_{I}$. The aim is thus twofold: (a) to identify the largest number of anomaly pixels in I; (b) to cover all the anomalous regions in I. An example of an image I containing anomalous elements, a binary mask of anomalies $\Omega_{I}$, and an approximated mask of anomalies $\tilde{\Omega_{I}}$ are represented in Figure 2.

The basic assumption for an automatic method for anomaly detection is that the method is trained with normal (i.e., anomaly free, see Figure 1a,b) images while it is tested with normal and anomalous images (see last Figure 1c,d). Hereinafter, we denote the set of normal images used for training the method as $I^{train}$, the set of normal images used for validating the method as $I^{val}$, while we denote the set of anomalous images used for testing the method as $I^{test}$. A method for anomaly detection learns a model of normality M(θ) from the training images $I^{train}$, where θ represents the free parameters of the model. The model M is inferred and used to assign an anomaly score z(x) to previously unseen test data x. Larger anomaly scores z(x) correspond to a higher abnormality with respect to the model of normality. The final classification of the test pattern is obtained by defining a threshold th such that the test pattern x is classified as “abnormal” if z(x)>th, or “normal” otherwise. The equation z(x)=th defines a decision boundary for the anomaly detection method [34]. The threshold th is found by exploiting the set $I^{val}$ that contains only images without anomalies.

## 4. Proposed Method

**Figure 3:**
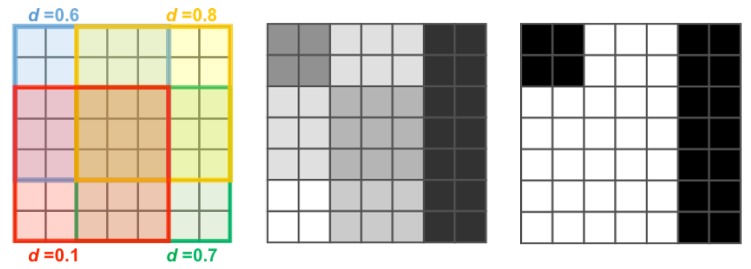
Simulated example of the map $\Theta_{I}$ and corresponding binary mask of anomalies $\tilde{\Omega_{I}}$. Here, $w_{t}=h_{t}=5$ and stride s=3 and the variable *d* represents the value of the average visual similarity between the local patch and the most similar subregions of the dictionary W.

The proposed anomaly detection method is region-based. The input image I is divided into a set of *T* subregions (or patches) of size $w_{t}\times h_{t}$ by following a regular grid sampling strategy with a stride *s*. The method takes each patch as input and it firstly computes its degree of abnormality and later it combines the response achieved on each subregion in order to have a map of anomalies $\Theta_{I}$ that is then thresholded to obtain the binary map ${\tilde{\Omega }}_{I}$. The degree of abnormality of a patch is obtained by computing the visual similarity between the given patch and a reference dictionary W of normal subregions taken from normal images belonging to $I^{train}$. The visual similarity is obtained by averaging the Euclidean distances between the feature vector extracted from a given subregion and the feature vectors extracted from the *m* most visually similar subregions of the dictionary. Each subregion is selected with a stride *s*, so each pixel of the image I may belong to different partially overlapping subregions. In this case, the degree of abnormality of each pixel is obtained by averaging the degree of abnormality of each corresponding subregion. Finally, the mask of anomalies $\tilde{\Omega_{I}}$ is obtained by thresholding $\Theta_{I}$ with th. Figure 3 shows an example of the map $\Theta_{I}$ obtained for $w_{t}=h_{t}=5$ and stride s=3 and the corresponding binary mask of anomalies $\tilde{\Omega_{I}}$.

### 4.1. Feature Extraction

A huge variety of features have been proposed in literature for describing image visual content. They are often divided into hand-crafted features and learned features. Hand-crafted features are extracted using a predefined strategy designed by an expert. Learned features are extracted using Convolutional Neural Networks (CNNs) [38,39].

CNNs are a class of learnable architectures adopted in many domains such as image recognition, image annotation, image retrieval, etc. [40]. CNNs are usually composed of several layers, each involving linear as well as nonlinear operators, that are learned jointly, in an end-to-end manner, to solve a particular tasks. A CNN architecture for image classification includes several convolutional layers followed by one or more fully connected layers. The output of the CNN is the output of the last fully connected layer. The number of output nodes is equal to the number of image classes [41].

A CNN that has been trained for solving a given task can be also adapted to solve a different task. It is not always possible to train an entire CNN from scratch because it is relatively rare to have a dataset of sufficient size. It is common to use a CNN that is pre-trained on a very large dataset (for instance, the ImageNet dataset, which contains 1.2 million images with 1000 categories [42]), and then use it either as an initialization or as a fixed feature extractor for the task of interest [43,44]. In the second case, the pre-trained CNN performs all the multilayered operations and, given an input image, the feature vector is the output of one of the last network layers [44]. The use of CNNs as a feature extraction method has demonstrated to be very effective in many pattern recognition applications [43,45,46,47].

The first notably CNN architecture that has showed very good performance upon previous methods on the image classification task is the AlexNet by Krizhevsky et al. [41] After the success of AlexNet, many other deeper architectures have been proposed such as: VGGNet [48], GoogleNet [49] and Residual Networks (ResNet) [50]. ResNet architectures demonstrated to be very effective on the ILSVRC 2015 (ImageNet Large Scale Visual Recognition Challenge) validation set with a top 1-recognition accuracy of about 80% [50].

In this paper, we use CNN-based features obtained by exploiting a deep residual architecture. Residual Network architectures are based on the idea that each layer of the network learns residual functions with reference to the layer inputs instead of learning unreferenced functions. Such architectures demonstrated to be easier to optimize and to gain accuracy by considerably increasing the depth [50]. For instance, the ResNet-50 architecture is about 20 times and eight times deeper than AlexNet and VGGNet, respectively.

In particular, the network architecture adopted in this paper is based on the ResNet-18 architecture, which represents a good trade-off between depth (that is computational time) and performance. The network architecture includes five convolutional stages (see Table 1 for further details). The network is pre-trained on the set of images defined by the ILSVRC 2015 challenge. The goal of this challenge is to identify the scene and object categories depicted in a photograph. The total number of categories is 1000. Although the network is pre-trained on scene and object images, it has demonstrated, in preliminary experiments, to work much better than a ResNet-18 pre-trained on texture images [47,51]. The visual appearance of textures is certainly more similar to the visual appearance of the SEM images considered in this paper. Notwithstanding this, the performance obtained by exploiting the texture-domain network are much worse than the performance obtained using a scene- and object-domain one. Actually, recognizing scenes and objects is more complicated than recognizing textures, and thus the network trained to recognize scenes and objects is more capable of recognizing unexpected anomalous patterns within SEM images.

Given the network, the output of a given layer is linearized to be used as a feature vector. We experiment with the use of two different layers of the network: the linearized output of the fifth convolutional stage (that is conv5_x) and the output of the average pooling layer (that is avgpool). The size of the feature vector is 25,088 (that is 7×7×512) in the case of the conv5_x layer and 512 in the case of the avgpool layer. The size of the feature vector affects the computational cost; then, the size is therefore reduced by applying dimensionality reduction techniques such as Principal Component Analysis.

### 4.2. Dictionary Building

The degree of abnormality of a patch is obtained by computing the visual similarity between the given patch and a reference dictionary W of normal subregions. The dictionary is built from the set of training images $I^{train}=\left\{I_{1},\cdots ,I_{L}\right\}$. For each image $I_{l}$, *T* patches $\left\{P_{1},\cdots ,P_{T}\right\}$ of size $w_{t}\times h_{t}$ are extracted following a regular grid and using a stride *s*. The total amount of patches extracted from the whole training set $I^{train}$ is $L_{T}=L\times T$. The feature extraction module computes for each patch $P_{t}$ a vector of features of size *N*, $f_{t}=\left\{f_{1}^{t},\ldots ,f_{N}^{t}\right\}$. The dimension of the feature vector is then reduced to *M* with M<N by applying the Principal Component Analysis (PCA) [52] to all the feature vectors extracted from $I^{train}$. *M* is the number of principal components such that a given percentage of the data variance is retained. For instance, if M=N, we have an exact approximation of the original data, and we say that 100% of the variance is retained. If M=0, we are approximating all the data with the zero-dimensional vector, and thus 0% of the variance is retained. For this work, we set a percentage of variance to 95%. After reduction, each feature vector is then normalized by using the following formula:

(2) $$f_{i}^{t}=\frac{f_{i}^{t}-\mu_{i}}{\sigma_{i}},\forall i\in \left(1,M\right),$$

where $\mu_{i}=\frac{1}{L_{T}}\sum_{t=1}^{L_{T}}f_{i}^{t}$ and $\sigma_{i}=\frac{1}{L_{T}}\sum_{t=1}^{L_{T}}{\left(f_{i}^{t}-\mu_{i}\right)}^{2}$. The normalization process makes all the feature components have zero mean and a unit variance.

**Figure 4:**
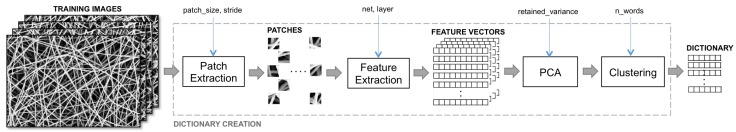
Examples of dictionary achieved considering different patch sizes and different number of subregions.

**Figure 5:**
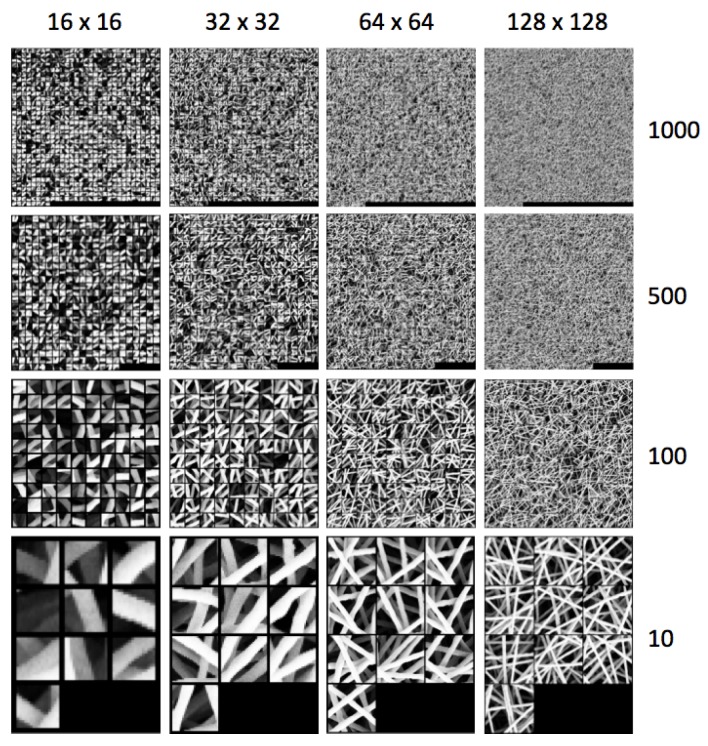
Examples of images corresponding to the features from the dictionary. Here, we show different dictionaries built with different patch sizes (16, 32, 64, 128) and number of clusters (10, 100, 500, 1000).

The dictionary is built by grouping all the reduced feature vectors of the training set into *k* clusters. We adopt the kmeans algorithm that takes the number of groups or clusters *k* as an input parameter and outputs the best *k* clusters and corresponding *k* centroids. A centroid is the mean position of all the elements of the cluster. For each cluster, we take the feature vector that is nearest to its centroid. The set of these *k* feature vectors compose the dictionary W. Figure 4 shows the pipeline for dictionary building. Figure 5 shows examples of dictionaries learned from images of the training set (anomaly free) with different patch sizes and number of clusters. The figure shows the subregions corresponding to each feature vector of the dictionary W.

### 4.3. Learning to Detect Anomalies

The rationale behind the proposed method is that, in order to detect anomalies within an image, we have to estimate how much a subregion of the image is far from being normal—in other words, how much it is anomalous. To do so, we have to learn from one or more examples of images without anomalies the concept of “normality”. We use *V* images from the validation set $I^{val}$ that are never used for the creation of the dictionary. Each image from $I^{val}$ is then processed as in the dictionary creation, that is, $V_{T}=V\times T$ patches are extracted from *V* images, the feature vectors of size *N* are extracted from the patches and then they are reduced to size *M* and finally they are normalized. At the end of this operation, the average of the Euclidean distances between all the feature vectors of the validation set and the *m* most similar subregions of the dictionary are calculated $d=\{d_{1},\cdots ,d_{V_{T}}\}$. The concept of “normality” is the model by the boundaries of a Gaussian function with mean and variance defined as follows: $\mu_{d}=\frac{1}{V_{T}}\sum_{t=1}^{V_{T}}d_{t}$ and $\sigma_{d}=\frac{1}{V_{T}}\sum_{t=1}^{V_{T}}{\left(d_{t}-\mu_{d}\right)}^{2}$. The boundaries allow to define a threshold to be used in testing time as:

(3) $$th=\mu_{d}+\alpha \sigma_{d},$$

where α being a positive real number that modulates the size of the boundaries. The smaller will be α, the more recall-oriented will be the system. In testing time, a subregion of an image is considered as anomalous if its average Euclidean distance $d_{test}$ with the *m* most similar subregions from the dictionary is higher than th.

## 5. Dataset Description

The dataset used in this work is composed of 45 images of nanofibrous materials acquired with a SEM (Scanning Electron Microscope). The external appearance of this material can be seen as a non-periodic continuous texture with intertwined filamentous elements that look like white wires, some examples with and without anomalies are in Figure 1. The dataset is composed of two disjoint subsets: a set of five images without anomalies, which we call “normal”, and a set 40 of images with defects that we call anomalies. All the defects have been manually annotated. The dataset and the defect annotations are publicly available at [53]. Each image I is gray-scale and of size 700×1024. The annotations associated with each anomalous image is a map $\Omega_{I}\in \left\{0,1\right\}$. Figure 2a,b shows an anomalous image along with its defect annotation. The set of normal images $I^{normal}$ is divided into two subsets $I^{train}$, $I^{val}$, which are, respectively, used to create the dictionary and to assess through visual self similarity the value of the threshold th used during the test time to convert the aggregated distance map $\Theta_{I}$ in the corresponding anomaly map $\tilde{\Omega_{I}}$. $I^{train}$ and $I^{val}$ have, respectively, a cardinality of 4 and 1. The set of test images $I^{test}$ contains 40 images with anomalies. For sake of comparison with the state-of-the-art, we use a subset made of 35 images obtained by removing images that are listed in $I^{test}$ at the following positions: 8, 15, 27, 31, 35. These images containing anomalies are required by the state-of-the-art methods to validate the parameters. Our method does not require to use images containing anomalies at any time apart from testing.

## 6. Experiments

**Figure 6:**
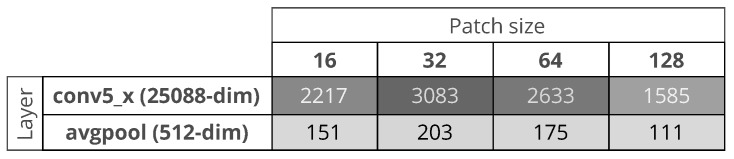
Dimension of the feature vectors after Principal Component Analysis reduction.

We experiment our method on SEM images of nanofibrous materials and we compare it with the method proposed by Carrera et al. [25]. We experiment several versions of our method by varying the following parameters:

1. patch size: 16×16, 32×32, 64×64, 128×128. The larger the patch is, the lower the computational time and the precision in defect localization;
2. dictionary size: 10, 100, 500, and 1000 numbers of subregions. The larger the number is, the higher is the time to calculate the similarity between a test patch and the subregions of the dictionary and the better the performance;
3. CNN layer output as feature vector: we use a ResNet-18 pre-trained on the images from ILSVRC 2015 (ImageNet Large Scale Visual Recognition Challenge) [54]. The input of the network is an RGB image of size 224×224. To adapt the input of the network to our problem, we up-sample the SEM image subregion to fit the network desired size and we convert the gray-scale SEM image to a RGB one by cloning the color channels. We take the output of the conv5_x of the network, which is a matrix 7×7×512. The output is linearized to be of size 25,088. Alternatively, we take the output of the average pooling layer (that we name avgpool). The 512-dimensional feature vector is obtained by linearizing the output matrix 1×1×512. All the feature vectors are L1 normalized;
4. Feature dimensionality reduction: the larger the size of the feature vector is, the higher is the time to calculate the similarity between a test patch and the subregions of the dictionary. In the case of PCA, we consider to take the first principal components such that the retained variance of the data is about 95%. Figure 6 shows, in the case of use of PCA, the reduced sizes of the feature vectors. The smallest feature vector is obtained by combining the avgpool with a patch size of 128×128, while the largest is obtained by combining the conv5_x with a patch size of 32×32.

For all the experiments, we use a stride *s* of eight pixels. The kmeans clustering algorithm is performed 10 times and the best output is taken in terms of intra cluster sum of squares. The kmeans algorithm uses the Euclidean distance, and it is initialized through the “kmeans++”, a procedure introduced by David Arthur et al. [55] to reduce the sensitivity of kmeans to the initialization seeds. The method is implemented in PyTorch [56], a Python-based tool for deep learning. The experiments are launched on an Ubuntu 16.04 Personal Computer equipped with an Intel i7-4790 CPU 3.60GHz (Santa Clara, California, U.S.) × 8, 16 GB RAM and a NVIDIA 1070 GPU (Santa Clara, California, U.S.).

### 6.1. Performance Metrics

The performance of the proposed method is evaluated by comparing the reference anomaly map $\Omega_{I}$ with the estimated anomaly map $\tilde{\Omega_{I}}$ obtained by thresholding the aggregated distance map $\Theta_{I}$. Each value of the threshold corresponds to an anomaly map; the smaller the threshold is, the lower will be the tolerance to which a sample is considered normal. As Carrera et al. [25] did in their work, we use two different evaluation procedures with the aim of evaluating two different aspects of the proposed method. The first one is the Receiver Operating Characteristic Curve (ROC) in which we evaluate the ability of the system to perform the per-pixel one-class classification. Specifically, we plot the true positive rate in a function of the false positive rate and we use as a performance index the Area Under Curve (AUC) to evaluate the global goodness of the method. Comparing each value $\tilde{p_{i}}\in \tilde{\Omega_{I}}$ with the relative ground truth value $p_{i}\in \Omega_{I}$, we define it as:

(4) $$\begin{array}{c} true\;negative\Leftrightarrow \tilde{p_{i}}=p_{i}=0, \end{array}$$

(5) $$\begin{array}{c} true\;positive\Leftrightarrow \tilde{p_{i}}=p_{i}=1, \end{array}$$

(6) $$\begin{array}{c} false\;positive\Leftrightarrow \tilde{p_{i}}=1\wedge p_{i}=0, \end{array}$$

(7) $$\begin{array}{c} false\;negative\Leftrightarrow \tilde{p_{i}}=0\wedge p_{i}=1. \end{array}$$

The true positive rate (TPR) and the false positive rate (FPR) is defined as follows:

(8) $$\begin{array}{c} TPR=\frac{TP}{TP+FN}, \end{array}$$

(9) $$\begin{array}{c} FPR=\frac{FP}{FP+TN}, \end{array}$$

where TP, FP, TN, FN are, respectively, the total number of true positives, false positives, true negatives and false negatives for each map $\tilde{\Omega_{I}}$. By varying the threshold value th, we obtain several values of TPR and FPR ranging from 0 to 1 and thus the ROC curve. The area under curve (AUC) is the performance index used to evaluate the global goodness of the method.

The second metric proposed by Carrera et al. [25] is the coverage percentage of defects. To this aim, we choose a threshold value th such that FPR is about 5%. To compute the defect coverage percentage, we detect each defect within each reference map $\Omega_{I}$ by finding each connected component $cc_{j}\in \Omega_{I}$. Each $cc_{j}$ represents a defect and for each defect we calculate the coverage factor as follows:

(10) $$\begin{array}{c} coverage\;factor_{j}=\frac{TP_{j}}{TP_{j}+FN_{j}}, \end{array}$$

where $TP_{j}$, $FN_{j}$ are, respectively, the true positives and the false negatives. In other words, the coverage factor of a connected component is the number of correctly detected pixels over its area.

### 6.2. Results

**Figure 7:**
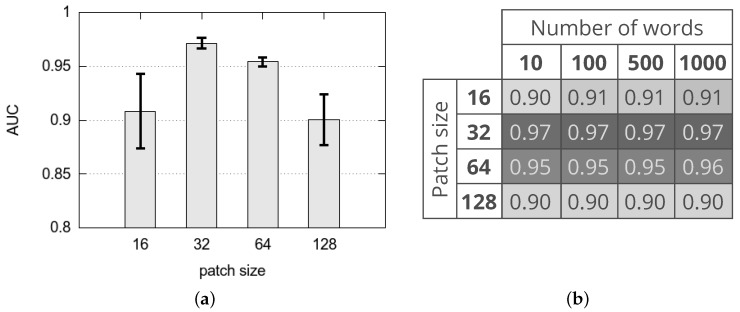
Area Under the Curve achieved with different variants of the proposed method. (**a**) average and standard deviation of the Area Under the Curve (AUC) achieved with different patch sizes whatever is the Convolutional Neural Network (CNN) layer adopted for feature extraction, whatever is the use of PCA to reduce the size of the feature vector and whatever is the number of words of the dictionary; (**b**) average of the AUC achieved with different patch sizes and number of words of the dictionary whatever is the CNN layer adopted for feature extraction and whatever is the use of PCA to reduce the size of the feature vector.

Figure 7 shows the AUC achieved with different variants of the proposed method. In particular, Figure 7a represents the average and standard deviation of the AUC achieved with different values of the patch size (that is 16, 32, 64 and 128) whatever is the CNN layer adopted for feature extraction (that is conv5_x and avgpool), whatever is the use of PCA to reduce the size of the feature vector and whatever is the number of words of the dictionary (that is 10, 100, 500, 1000). Results suggest that, in terms of AUC, the best performing variants are obtained with patch size 32 whatever are the other parameters, while the worst variants are with patch size 128 and 16. It should be noticed that the best variants achieve an average AUC of about 97% while the worst variants achieve an average AUC of about 90%. For comparison, we select the method proposed by Carrera et al. [25] that is both the most performing and the most recent state-of-the-art methods on this dataset. It achieves an AUC of about 92%.

Figure 7b shows the average of the AUC achieved with different patch sizes and number of words of the dictionary whatever is the CNN layer adopted for feature extraction and whatever is the use of PCA to reduce the size of the feature vector. Results suggest that, in terms of AUC, the best performing variants are obtained combining a patch size 32 with all the possible sizes of the dictionary. From this result, it is quite clear that the best solution is based on a patch size of 32×32 pixels and size of the dictionary equals 10.

**Figure 8:**
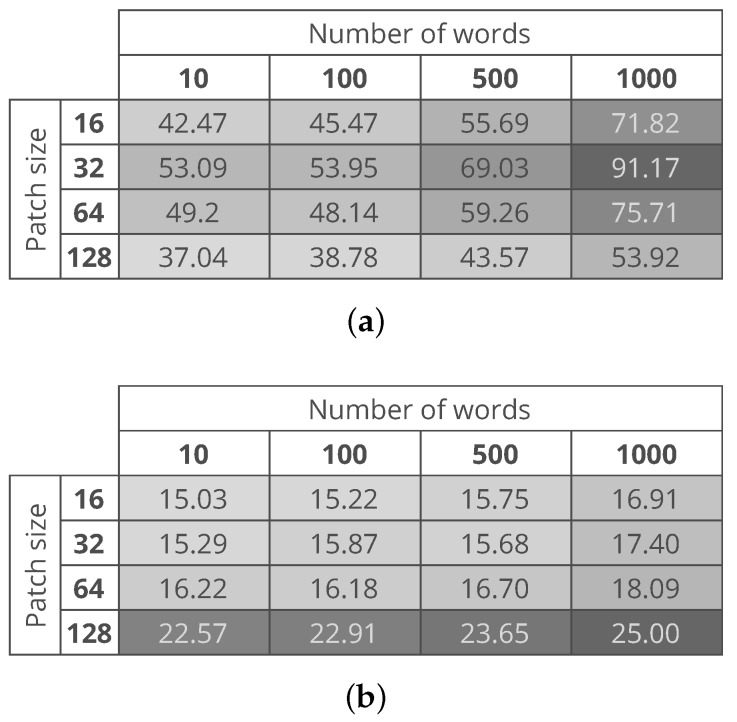
Average time to process a test image. (**a**) time needed in the case of features extracted from the conv5_x of the CNN; (**b**) time needed in the case of features extracted from the avgpool of the CNN.

Figure 8a,b show the computational time needed to process an entire image with the use of conv5_x and avgpool CNN layers, respectively. The figure shows that the variants with a lower computational cost are the ones based on features extracted from the avgpool layer. This is related to the fact that whether we the PCA or not, the size of the feature vector extracted from the conv5_x layer is five times larger than the size of the feature vector extracted from the avgpool layer. The best variants of the proposed method are the ones that consider a patch size 32×32 and a dictionary of 10 words. The proposed method variants take about 53 s and 15 s in the case of conv5_x and avgpool respectively measured on the same machine. As argued by Carrera et al. [25], such a computational time makes it possible to monitor the production process of the nanofibers.

**Figure 9:**
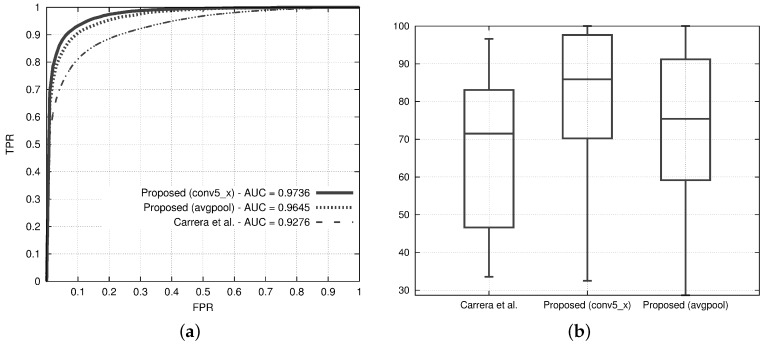
Results from the two variants of the proposed method, one with conv5_x and the other with avgpool, and comparison with the method proposed by Carrera et al. [25]. Both the variants consider the PCA to reduce the feature vector, a patch size of 32 pixels and a dictionary of size 10. (**a**) Receiver Operating Characteristic (ROC) curves. For each ROC curve, the corresponding AUC values are in the legend; (**b**) box-plots reporting the distribution of the defect coverage obtained at a fixed False Positive Rate (FPR) = 5%.

Figure 9a,b show the ROC curves and the defect coverage box plots of the best variants of the proposed method and the state of the art. The ROC curves of both the variants of the proposed method are higher than the state of the art, and the AUC of both variants is about 5% higher than the state of the art. It is quite interesting to note that the variant with the avgpool layer achieves almost the same AUC as the one with the conv5_x while having a computational time that is about three times less than the computational time of the conv5_x-based one.

The box plots of Figure 9b show that the state-of-the-art solution achieves an average value of defect coverage that is quite similar to the worst value obtained by the best variant of the proposed method, which covers at least the 50% of the anomalies for more than 85% of their area.

**Figure 10:**
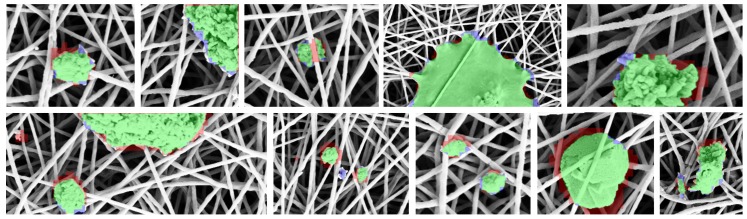
Closeup of the anomalies found by the proposed method. True positives, false positives, and false negatives are showed, respectively, as green, red and blue color. For visualization purposes, the images are slightly cropped and scaled to focus on fine- and coarse-grained anomalies.

Figure 10 shows some defect detections and localizations obtained with a variant of the proposed method that use conv5_x layer, patch size 32, PCA for dimensionality reduction and a dictionary of size 10. The images are close-ups of fine- and coarse-grained defects. True positives are green colored, false positives are red colored, while false negatives are blue colored. It is quite evident that the proposed method is quite accurate to detect coarse grained defects, and it should be further improved to detect medium- and fine-grained defects.

## 7. Conclusions

In this paper, we propose a computer-vision-based method for the detection and localization of anomalies in Scanning Electron Microscope images of nanofibrous materials. The automatic detection and localization of defects in the production process of nanofibers is a crucial activity that reduces the manufacturing cost. The proposed method detects defects as anomalies with respect to “normal” patterns. The method is patch-wise and is based on a measure of visual similarity between a subregion of the test image and the subregions of a dictionary representing “normal” nanofibers. The lower the visual similarity, the higher the degree of abnormality. A subregion contains a defect if its degree of abnormality is outside a boundary of normality built by computing the visual self similarity between normal subregions. The method exploits Convolutional Neural Networks for describing the visual content of each subregion.

The method has been designed and tested on SEM images of nanofibrous materials. However, we strongly believe that our approach could be fruitfully employed in other domain—for example, for Transmission Electron Microscope images.

The two variants of the proposed method, benchmarked on a publicly available dataset of SEM images, outperform the state of the art of about 5% by reaching an area under the curve of about 97%. The variants take about 50 s and 15 s respectively to process a test image of 700×1024 on an Ubuntu 16.04 Personal Computer equipped with an Intel i7-4790 CPU 3.60GHz × 8, 16 GB RAM and a NVIDIA 1070 GPU. This computational time, as argued by Carrera et al. [25], is less than the time employed to produce a nanofiber sample.

Experimental results show that the proposed method is quite accurate to detect coarse-grained defects, while it is less accurate to detect medium- and fine-grained ones. Future investigations could include the processing of the SEM images at different scales in order to be more accurate to detect small anomalies.

## Figures and Tables

**Table 1:** ResNet-18 Architecture.

Layer Name	Output Size	ResNet-18
conv1	112×112×64	7×7, 64, stride 2
conv2_x	56×56×64	3×3 max pool, stride 2
		$\left[\begin{array}{c} 3\times 3,64\\ 3\times 3,64 \end{array}\right]$× 2
conv3_x	28×28×128	$\left[\begin{array}{c} 3\times 3,128\\ 3\times 3,128 \end{array}\right]$× 2
conv4_x	14×14×256	$\left[\begin{array}{c} 3\times 3,256\\ 3\times 3,256 \end{array}\right]$× 2
conv5_x	7×7×512	$\left[\begin{array}{c} 3\times 3,512\\ 3\times 3,512 \end{array}\right]$× 2
average pool	1×1×512	7×7 average pool
fully connected	1000	512×1000 fully connections
softmax	1000	
